# Algorithmically defined therapeutic targets: integrating single-cell transfer learning frameworks with small molecule drugs to reverse disease-associated cell fates

**DOI:** 10.3389/fphar.2026.1786561

**Published:** 2026-03-25

**Authors:** Xiaofeng Ma, Zhuo Zuo, Wei Shi, Yulong Sun

**Affiliations:** 1 Key Laboratory for Space Biosciences & Biotechnology, School of Life Science and Technology, Institute of Special Environmental Biophysics, Research Center of Special Environmental Biomechanics and Medical Engineering, Engineering Research Center of Chinese Ministry of Education for Biological Diagnosis, Treatment and Protection Technology and Equipment, Northwestern Polytechnical University, Xi’an, Shaanxi, China; 2 Emergency Center, The Second Hospital of Lanzhou University, Lanzhou, Gansu, China; 3 Key Laboratory of Gansu Province for Urological Diseases, Institute of Urology, Gansu Urological Clinical Center, The Second Hospital of Lanzhou University, Lanzhou, China

**Keywords:** deep transfer learning, drug resistance, pharmacological reprogramming, single-cell sequencing, tumor microenvironment

## Abstract

The high heterogeneity of the disease microenvironment is a critical factor contributing to therapeutic failure and the emergence of drug resistance; however, predicting drug responses with precision at single-cell resolution remains a substantial challenge. Traditional pharmacogenomic studies are constrained by averaged signals at the population level, which frequently obscure rare yet lethal resistant subpopulations. This article reviews a closed-loop strategy that integrates computational pharmacology with cell biology to address this dilemma. First, we explore computational frameworks based on Deep Transfer Learning and Domain Adaptation, such as scDEAL and SCAD. These algorithms can transfer pharmacological knowledge from large-scale cell lines to clinical single-cell data, thereby enabling virtual prediction of cellular drug sensitivity in the absence of experimental labels. Second, based on algorithmic predictions, we elucidate chemotherapy-induced Transcriptional Stress States and their co-evolutionary mechanisms with inflammatory stromal cells, as well as interactions that construct an immunosuppressive barrier protecting residual disease. Finally, we demonstrate the feasibility of reprogramming these specific pathological states using small-molecule drugs (e.g., decitabine, benzofuran derivatives), including the reversal of macrophage polarization imbalance in spinal cord injury and the amelioration of osteogenic differentiation disorders in osteoporosis. This integrated “algorithm prediction–mechanism elucidation–drug intervention” strategy provides a novel paradigm for precision therapy to reverse disease-associated cell fates.

## 1 Introduction

The extensive heterogeneity of the disease microenvironment represents one of the core challenges facing modern biomedicine, a phenomenon particularly pronounced in malignant tumors, neural injuries, and metabolic bone diseases. Although single-cell RNA sequencing (scRNA-seq) technology has dramatically improved the resolution of cellular atlases—revealing previously unknown subpopulations and transcriptional states—it continues to face a critical bottleneck in drug development: the lack of large-scale, high-throughput drug-response labels (Ground Truth) at the single-cell level. Current drug screening predominantly relies on cell lines or bulk tissue samples; such averaged signals often mask the pharmacological characteristics of rare subpopulations or cells in specific functional states that play decisive roles within the tissue. For instance, in studies of pancreatic ductal adenocarcinoma (PDAC), despite the known complexity of the microenvironment, traditional analytical methods struggle to precisely dissect the dynamic evolution of distinct stromal cell subpopulations under chemotherapy pressure and their specific contributions to drug sensitivity (Chen K. et al., 2023). Consequently, accurately predicting the sensitivity of individual cells in clinical samples to particular drugs, in the absence of direct experimental validation, has become an urgent problem in precision medicine.

To overcome this data sparsity challenge, a new paradigm has recently emerged in the field of computational biology: utilizing Deep Transfer Learning (DTL) and Domain Adaptation (DA) algorithms to transfer and map massive amounts of drug sensitivity data accumulated in traditional pharmacogenomic databases (e.g., Genomics of Drug Sensitivity in Cancer (GDSC) and Cancer Cell Line Encyclopedia (CCLE)) onto clinical single-cell transcriptomic data. For example, the scDEAL computational framework constructs deep neural networks. It uses the Maximum Mean Discrepancy (MMD) loss function to eliminate distributional differences between *in vitro* cell lines (source domain) and *in vivo* microenvironments (target domain), thereby enabling virtual annotation of single-cell drug responses (Chen et al., 2022). Similarly, the SCAD model introduces Adversarial Domain Adaptation strategies, further enhancing the accuracy of transferring pharmacological knowledge across different data distributions, enabling researchers to identify pre-existing resistant subpopulations at single-cell resolution (Zheng et al., 2023). Furthermore, deep learning-based deconvolution algorithms, such as HASCAD, link high-resolution cellular composition information to survival prognosis in large clinical cohorts, providing a powerful computational engine for understanding the pharmacological responses of complex tissues (Chiu et al., 2023).

Through the lens of these computational tools, researchers are beginning to re-examine the biological nature of therapeutic failure. Increasing evidence suggests that residual disease following chemotherapy or targeted therapy is not merely a selection of genetically mutant clones, but also involves complex reprogramming of transcriptional states and microenvironmental co-evolution. For instance, following neoadjuvant therapy in ovarian cancer, tumor cells often enter a specific “Transcriptional Stress State”, characterized by high expression of inflammatory factors and restricted proliferation, accompanied by the enrichment of inflammatory cancer-associated fibroblasts (iCAFs), which collectively construct a niche supporting cell survival (Zhang K. et al., 2022). Similarly, in pancreatic cancer, chemotherapy-induced pressure significantly increases activated pancreatic stellate cells (Activated PSCs), which encase the tumor to form a physical barrier, accompanied by T cell exhaustion and the loss of immune checkpoint receptor-ligand pairs, leading to resistance to immunotherapy (Chen K. et al., 2023). This “stress-stroma” adaptive network, defined with algorithmic assistance, offers a new perspective beyond genomics for understanding the acquisition of drug resistance.

**FIGURE 1:**
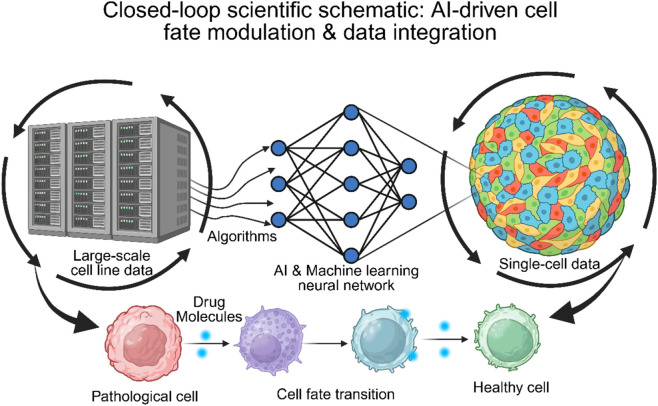
A Novel Paradigm for Precision Medicine Integrating Deep Transfer Learning and Pharmacological Reprogramming. (1) Data Gap and Computational Bridge: The left panel displays the Source Domain (e.g., GDSC/CCLE cell line databases) with extensive drug response labels. In contrast, the right panel shows the Target Domain (clinical scRNA-seq data) lacking drug labels but possessing high biological heterogeneity. A computational bridge is constructed via Deep Transfer Learning and Domain Adaptation algorithms to align feature distributions. (2) Biological Mechanism Elucidation: Algorithmic predictions reveal chemotherapy-induced Transcriptional Stress States and “Stromal Barriers” within the microenvironment. (3) Pharmacological Intervention and Reprogramming: Based on mechanistic insights, small molecules (e.g., Decitabine, Benzofuran derivatives) are deployed to target specific transcriptional programs, reprogramming pathological cells (e.g., M1 macrophages, stressed tumor cells) into therapeutically favorable phenotypes (e.g., M2 macrophages, apoptotic cells). (4) Closed-Loop Validation: An iterative cycle from virtual screening to wet-lab validation (*in vitro*/*in vivo*).

Based on the precise identification of the aforementioned pathological states, therapeutic strategies are shifting from simple cellular cytotoxicity to the “reprogramming” of cell fate. Using small-molecule drugs to target specific epigenetic or signaling pathways may reverse disease-associated cell fates. For example, Decitabine has been proven to reprogram pro-inflammatory macrophages (M1) in the spinal cord injury microenvironment into an anti-inflammatory repair phenotype (M2) through demethylation, thereby significantly promoting neural regeneration (Zhang Q. et al., 2023); meanwhile, novel benzofuran derivatives can correct the osteoblast/osteoclast balance dysregulation in senile osteoporosis by modulating the BMP-2/ATF4 signaling axis, promoting bone formation (Zhou et al., 2023). Additionally, studies of the human developmental immune atlas have provided clues for identifying new therapeutic targets. For instance, newly discovered proangiogenic macrophages (PraM) and peripheral microglia-like cells regulate tissue remodeling through specific transcription factors, suggesting that pharmacological intervention in these specific subpopulations could promote tissue regeneration (Wang et al., 2023). Compared with existing reviews that predominantly focus either on computational benchmarking or on the biological characterization of the tumor microenvironment (TME), the novelty of this review lies in proposing an interdisciplinary, closed-loop translational framework. This framework bridges the gap between *in silico* algorithms and experimental pharmacology, shifting the therapeutic paradigm from simple cytotoxicity toward pharmacological reprogramming (Figure 1). Furthermore, by extending this paradigm beyond oncology to regenerative medicine, we provide a uniquely comprehensive and actionable roadmap for utilizing AI-driven single-cell insights to guide drug discovery across diverse pathologies. This article systematically reviews the closed-loop strategy from “algorithm prediction” to “mechanism elucidation” and finally to “drug intervention”, exploring how to integrate single-cell transfer frameworks with small-molecule drugs to achieve precise reversal of disease-associated cell fates.

## 2 Computational engines: mapping population pharmacology to single-cell resolution

While scRNA-seq offers high-resolution insights into cellular heterogeneity, the lack of direct drug-response labels in clinical data limits its utility for precision screening (Chen et al., 2022). To bridge this genotype-phenotype gap, computational biology has integrated DTL and network pharmacology to map population-level pharmacological laws onto single cells (He Y. et al., 2025).

### 2.1 Foundations of transfer learning and Domain Adaptation

Before delving into specific single-cell applications, it is essential to establish the foundational concepts of Transfer Learning (TL) and DA. In classical machine learning, a model trained on one dataset often performs poorly on another due to differing data distributions. TL is a paradigm designed to overcome this by transferring knowledge learned from a data-rich source domain to a related but data-scarce target domain.

However, when the statistical distributions of the source and target domains differ significantly—a phenomenon known as “domain shift”—the efficacy of traditional TL drops. DA emerged as a specific subfield of TL to align these divergent distributions. Classical DA models achieve this through two main mathematical strategies. The first involves using deep Autoencoders combined with statistical distance metrics, such as MMD, to penalize and minimize distributional differences between domains in a latent feature space. The second prominent strategy employs Generative Adversarial Networks (GANs), such as Adversarial Discriminative Domain Adaptation (ADDA). In this classical model, a feature extractor and a domain discriminator are trained adversarially: the extractor attempts to learn representations that can “fool” the discriminator into failing to distinguish the data’s origin, thereby yielding “domain-invariant” features. In recent years, these foundational computer science concepts have been ingeniously adapted to bioinformatics to bridge the gap between *in vitro* pharmacogenomics and *in vivo* single-cell transcriptomics.

### 2.2 Deep transfer learning and Domain Adaptation

**FIGURE 2:**
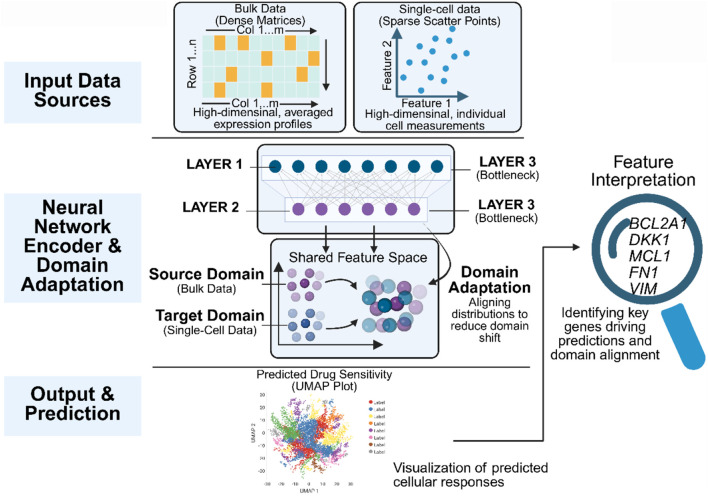
Architecture of the Deep Transfer Learning Framework Bridging the Genotype-Phenotype Gap. This figure illustrates the core working principles of models such as scDEAL and SCAD. (1) Feature Extraction and Denoising: Deep Denoising Autoencoders (DAE) process Source Domain (Bulk RNA-seq) and Target Domain (scRNA-seq) data separately to extract latent low-dimensional genomic features while removing scRNA-seq-specific dropout noise. (2) Adversarial Domain Adaptation (ADDA): A Discriminator is introduced for adversarial training with the Feature Extractor. The Discriminator attempts to distinguish the data source. At the same time, the Extractor tries to “deceive” the Discriminator, ultimately generating “Domain-invariant Features” that achieve perfect alignment of the Source and Target domains in high-dimensional space. (3) Drug Sensitivity Prediction and Interpretation: Aligned features are input into a Predictor to output the drug sensitivity probability for every single cell. Integrated Gradients are used to backpropagate and identify key Signature Genes that determine drug resistance.

The primary strategy involves transferring drug sensitivity data from large-scale cell line databases (Source Domain) to clinical single-cell data (Target Domain). To overcome the significant “Domain Shift” between *in vitro* models and *in vivo* tissues, algorithms must align feature distributions (Zheng et al., 2023). For instance, the scDEAL framework uses Deep Denoising Autoencoders and MMD to align gene features across domains, enabling virtual drug-response prediction while preserving biological heterogeneity (Chen et al., 2022). Similarly, the SCAD model employs Adversarial Domain Adaptation strategies to generate domain-invariant features, facilitating the identification of pre-existing resistant subpopulations in untreated samples, as demonstrated in melanoma and head and neck cancer (Zheng et al., 2023). It is important to note that cell line databases inherently lack the complex stromal and immune components found in clinical tissues. Current DTL algorithms primarily address this lack of biological context by isolating domain-invariant intrinsic molecular signatures (Chen et al., 2022; Zheng et al., 2023). Consequently, these models predict the intrinsic drug response potential of tumor cells. To account for extrinsic, microenvironment-driven drug resistance, researchers increasingly couple these predictions with cell-cell communication tools (e.g., sc2MeNetDrug) or deconvolution algorithms (e.g., HASCAD) to reconstruct interactions between tumor cells and TME components in the target clinical data (Chiu et al., 2023; Feng et al., 2024). To ensure that the ‘domain-invariant features' generated in the bottleneck layer represent biologically meaningful pathways (e.g., apoptosis or DNA repair) rather than merely correcting for technical batch effects, modern DTL frameworks incorporate interpretability modules. For instance, scDEAL utilizes Integrated Gradients for backpropagation, mapping abstract latent features back to the original input space to identify specific ‘signature genes' driving the predictions. Subsequent pathway enrichment analyses have confirmed that these extracted genes are highly enriched in classical resistance pathways, including DNA repair and regulation of apoptosis (Chen et al., 2022). Similarly, the adversarial training mechanism in SCAD forces the network to discard domain-specific background noise, thereby isolating shared biological determinants across domains (Zheng et al., 2023) (Figure 2).

### 2.3 Feature Extraction and deep deconvolution

Precise target identification requires mitigating scRNA-seq noise (e.g., dropout events) and patient-specific effects (Gezelius et al., 2024). Algorithms like PRIMUS employ Bilinear Poisson Regression to decompose background noise, revealing “Latent Phenotypic Clusters” such as chemotherapy-selected stress states across patients (Zhang K. et al., 2022). Furthermore, integration tools like Harmony help construct reference atlases for deep learning-based deconvolution models such as HASCAD. This approach links high-resolution cellular composition to clinical survival prognosis, providing a computational basis for patient stratification (Chiu et al., 2023) (Shi et al., 2024; He C. et al., 2025).

### 2.4 Network pharmacology and molecular targeting

To pinpoint druggable targets, tools like sc2MeNetDrug integrate single-cell differentially expressed genes with protein-protein interaction networks to identify “Hub genes” critical for signal transduction (e.g., *Vav1*, *Itgb5*) (Feng et al., 2024). Subsequent molecular docking simulations (e.g., using AutoDock) validate the binding efficacy of small molecules to these targets. This workflow has successfully identified agents such as Decitabine for macrophage reprogramming (Zhang Q. et al., 2023) and DB03742 for osteoporosis treatment (Zhang Z. et al., 2022), thereby completing the loop from transcriptional features to candidate drugs (Zhou et al., 2023). Crucially, while computational engines like DTL provide high-throughput virtual screening, their predictions must always be anchored by rigorous biological interpretation and wet-lab validation. Rather than treating these models as black boxes, researchers must translate the extracted mathematical features into actionable biological mechanisms—such as mapping latent resistance signatures to specific biological pathways (e.g., the anti-apoptotic gene BCL2A1 or metabolic reprogramming). Furthermore, the closed-loop strategy mandates that all *in silico* predictions undergo orthogonal validation through *in vitro* assays (e.g., fluorescence-activated cell sorting (FACS), multiplex immunohistochemistry) and *in vivo* animal models. This rigorous validation step confirms the actual pharmacological efficacy of small-molecule reprogramming, ensuring that algorithmically defined targets are genuinely druggable.

To assist pharmacologists in selecting the most appropriate framework for their specific translational pipelines, a comprehensive comparison of these computational tools—highlighting their algorithmic mechanisms, strengths, limitations, and ideal scenarios—is summarized in Table 1.

**TABLE 1:** Comparative analysis of single-cell computational frameworks for drug discovery.

Framework	Core algorithm/Mechanism	Required input data types	Strengths	Limitations	Ideal application scenarios	References
scDEAL	Deep Denoising Autoencoder + Maximum Mean Discrepancy (MMD)	Source: Bulk RNA-seq (Cell lines)Target: scRNA-seq (Clinical)	Preserves biological heterogeneity while effectively mitigating scRNA-seq dropout noise	Does not inherently account for spatial architecture or direct stromal-tumor interactions	Virtual drug screening and predicting intrinsic drug response at single-cell resolution on dissociated clinical samples	Chen et al. (2022)
SCAD	Adversarial Discriminative Domain Adaptation (ADDA)	Source: Bulk RNA-seq (Cell lines)Target: scRNA-seq (Clinical)	Stronger alignment of highly divergent distributions via adversarial training; robust feature extraction	Adversarial training can be mathematically unstable and computationally intensive	Identifying pre-existing, rare drug-resistant subclones in treatment-naïve patient samples across highly divergent datasets	Zheng et al. (2023)
HASCAD	Deep neural network-based hierarchical deconvolution	Reference: scRNA-seqTarget: Bulk RNA-seq (Clinical cohorts)	Links high-resolution single-cell signatures to large-scale clinical survival and prognosis data	Relies heavily on the quality and completeness of the predefined scRNA-seq reference atlas	Patient stratification and evaluating how cellular composition impacts bulk tissue pharmacological response	Chiu et al. (2023)
sc2MeNetDrug	Differentially Expressed Genes (DEGs) + Protein-Protein Interaction (PPI) Networks	scRNA-seq (Target cells and Microenvironmental cells)	Uncovers inter-cell signaling targets and identifies repurposable small molecules via virtual molecular docking	Requires well-annotated receptor-ligand databases; computationally inferred PPIs demand rigorous *in vitro* validation	Discovering drugs that disrupt pathological cell-cell communication (e.g., tumor-stroma crosstalk and immune evasion)	Feng et al. (2024)

## 3 Biological entities: chemotherapy-induced stress and subclonal evolution

The combination of scRNA-seq and deep transfer learning algorithms enables us to penetrate the surface of clinical treatment responses and to analyze the dynamic, evolutionary trajectories of tumor cells under drug pressure. This process reveals a core biological fact: the emergence of drug resistance is not driven solely by the random selection of genomic mutations, but relies intensely on the phenotypic transition of tumor cells towards a specific “Transcriptional Stress State”, as well as the immune escape mechanisms triggered thereby (Zhang K. et al., 2022).

### 3.1 Subclonal selection and enrichment of transcriptional stress states

Under the intense selective pressure of chemotherapy drugs, tumor cells exhibit high plasticity. Through longitudinal scRNA-seq analysis of paired samples from High-Grade Serous Ovarian Cancer (HGSOC) patients before and after neoadjuvant chemotherapy, researchers found that while chemotherapy effectively cleared most actively proliferating cancer cells, it did not completely eradicate the tumor. Instead, it significantly enriched a class of pre-existing “stress-associated” subclones (Zhang K. et al., 2022). These residual cells characteristically expressed high levels of Immediate Early Genes (IEGs), including *FOS*, *JUN*, *IL6*, *TNF*, and anti-apoptotic genes such as *MCL1*, exhibiting characteristics of low proliferation but high survival. Notably, this “stress state” was not newly generated post-treatment but existed as a pre-existing transcriptional program that became the dominant subpopulation under chemotherapy selection pressure, and its abundance was significantly negatively correlated with patients' Progression-Free Survival (PFS) (Zhang K. et al., 2022). It is important to acknowledge that high IEG expression can often arise as a technical artifact during the enzymatic tissue dissociation step of scRNA-seq. To confidently differentiate genuine chemotherapy-induced stress from *ex vivo* technical noise, the reviewed studies employed rigorous validation strategies. These include using strict longitudinal matched-control designs to control for baseline dissociation noise, applying computational deconvolution algorithms (e.g., PRIMUS) to regress out technical variance, and, most crucially, conducting orthogonal *in situ* validation. By performing multiplex immunohistochemistry on intact, undissociated tissue sections, researchers confirmed that these transcriptional stress states are not dissociation artifacts but *bona fide* biological entities driving microenvironmental resistance post-chemotherapy (Zhang K. et al., 2022).

**FIGURE 3:**
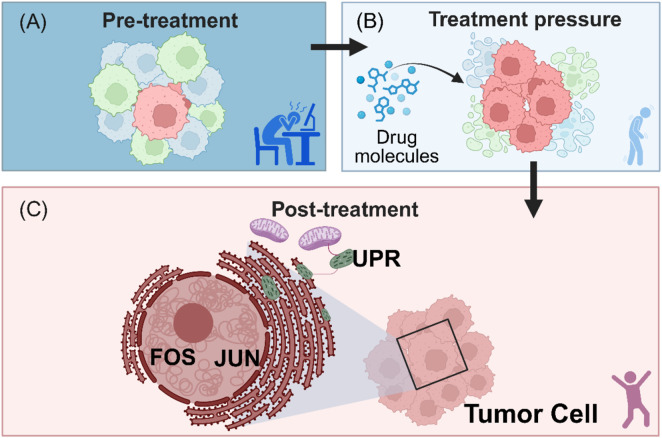
Cellular State Plasticity and Enrichment of Resistant Subpopulations Under Therapeutic Pressure. **(A)** Subclonal Selection Model: Illustrates changes in tumor clonal structure pre- and post-chemotherapy. Rare subclones (red) present before treatment are significantly enriched after treatment, while sensitive subclones (blue) are eliminated. **(B)** Transcriptional Stress Signatures: Resistant subpopulations are not dormant but exhibit a distinct “Transcriptional Stress State”, characterized by high expression of immediate-early genes (e.g., FOS, JUN) and inflammatory factors (IL6, TNF). **(C)** Metabolic and Signaling Reprogramming: Depicts the metabolic shift (e.g., from oxidative phosphorylation to glycolysis) and activation of the Unfolded Protein Response (UPR) within resistant cells, adaptive changes that support cell survival under drug pressure.

Similar phenomena have been observed in neoadjuvant therapy (chemotherapy combined with anti-PD-1 therapy) for PDAC. scRNA-seq analysis of resected residual lesions identified a unique proliferative tumor subpopulation (Subcluster 6) that not only highly expressed cell-cycle and related genes (e.g., MKI67, PCNA, TOP2A) but also specifically upregulated the stemness markers ASPM and the epigenetic regulator *EZH2* (Chen K. et al., 2023). More critically, this subpopulation exhibited significantly elevated Unfolded Protein Response (UPR) scores and glycolytic metabolic features, suggesting that tumor cells adapt to therapeutic pressure through metabolic reprogramming and enhanced stress tolerance (Chen K. et al., 2023) (Figure 3).

Recent multiplexed single-cell pharmacological transcriptomics studies on HGSOC have further revealed drug-induced feedback loops. Dini et al., utilizing antibody-oligonucleotide conjugates for multiplexed scRNA-seq screening, discovered that PI3K/AKT/mTOR inhibitor treatment leads to compensatory activation of Receptor Tyrosine Kinase (RTK) pathways, particularly *CAV1*-mediated *EGFR* upregulation; this “drug-induced adaptability” constitutes another molecular basis for resistance (Dini et al., 2025).

### 3.2 Co-evolution of immune escape and exhaustion mechanisms

Chemotherapy-induced stress states not only alter the fate of tumor cells themselves but also profoundly reshape the immune landscape within the microenvironment, leading to the emergence of “immune exclusion” or “immune exhaustion” phenotypes. In studies of Multiple Myeloma (MM), Chen et al. found that VCD regimens (Bortezomib-Cyclophosphamide-Dexamethasone) caused a drastic rearrangement of transcriptional programs in tumor cells: original metabolism-related programs were downregulated, while “stress-associated” programs were significantly upregulated. Notably, the specific transcription factor *YBX1* played a key role in mediating immune escape. High expression of *YBX1* was positively correlated with downregulation of MHC Class I molecules (HLA-A/B/C); this transcriptional regulatory mechanism directly impaired immune surveillance, leading to reduced infiltration and functional exhaustion of CD8^+^ T cells and NK cells (Chen M. et al., 2023).

In residual lesions of solid tumors, this immunosuppressive microenvironment is even more complex. Residual lesions of PDAC after neoadjuvant therapy exhibited distinct features of immune checkpoint loss: there was a lack of effective receptor-ligand interactions (e.g., extremely low PD-1/PD-L1 expression) between tumor cells and T cells, which may explain their insensitivity to subsequent Immune Checkpoint Inhibitor (ICI) therapy (Chen M. et al., 2023). Simultaneously, T cells in residual lesions exhibited an exhausted state (Tex), characterized by high expression of LAG3 and TIGIT. At the same time, macrophages tended to express SPP1, suggesting polarization towards a pro-tumor M2 phenotype (Chen M. et al., 2023).

For leukemia patients receiving Donor Lymphocyte Infusion (DLI) therapy, single-cell analysis revealed an association between IFN-γ response in the bone marrow microenvironment and relapse risk. Patients who relapsed after treatment exhibited downregulation of MHC Class II molecules and upregulation of co-inhibitory receptor ligands in leukemia cells, indicating that tumor cells escape alloimmune pressure by reducing immunogenicity (Maurer et al., 2025). Furthermore, studies targeting Myeloid-Derived Suppressor Cells (MDSCs) found that while Bruton’s Tyrosine Kinase (BTK) inhibitors could downregulate pro-inflammatory factors like *CXCL8* in MDSCs, their effects exhibited significant heterogeneity across patients, further emphasizing the necessity of resolving the immune microenvironment at single-cell resolution for predicting efficacy (Savardekar et al., 2024).

To accurately parse these complex cell states and interaction networks, the application of computational tools is crucial. For instance, the scDEAL model identified key resistance genes enriched in “DNA repair” and “negative regulation of cell death” pathways (e.g., *BCL2A1*, *DKK1*) in cisplatin-treated oral squamous cell carcinoma cells (Chen et al., 2022), while the SCAD model successfully predicted pre-existing resistant subpopulations (Zheng et al., 2023). These findings collectively depict a dynamically evolving resistance ecosystem in which stress adaptation in tumor cells and inhibitory remodeling of the immune microenvironment are mutually causal.

## 4 Microenvironmental niches: stromal barriers and novel subpopulations

The refractory nature of tumors and injured tissues is often attributed to their unique microenvironmental niches. These niches not only provide sanctuary for pathological cells but also lock in disease states through complex intercellular communication networks. scRNA-seq, combined with high-resolution developmental atlases, is redefining our understanding of stromal cell heterogeneity and myeloid cell functional subpopulations, revealing multidimensional regulatory mechanisms ranging from “inflammatory barriers” to “angiogenic niches” (Wang et al., 2023).

### 4.1 Co-evolution of inflammatory stroma and barrier construction

**FIGURE 4:**
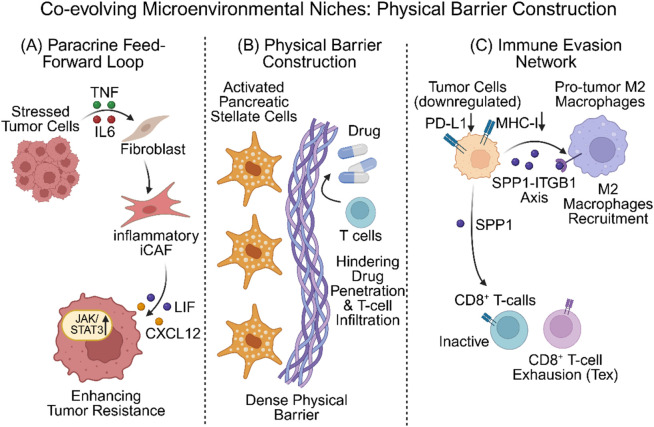
Co-evolving Microenvironmental Niches: Physical Barriers and Immune Exclusion. **(A)** Paracrine Feed-Forward Loop: Stressed tumor cells secrete TNF/IL6, inducing fibroblasts to transform into an inflammatory subtype (iCAF). In turn, iCAF secretes LIF/CXCL12, thereby enhancing tumor cell resistance (via JAK/STAT3 pathway activation). **(B)** Physical Barrier Construction: Activated Pancreatic Stellate Cells and collagen deposition form a dense physical barrier, hindering drug penetration and T-cell infiltration. **(C)** Immune Evasion Network: Illustrates a “cold tumor” microenvironment. Tumor cells downregulate PD-L1 and MHC-I while recruiting pro-tumor M2 macrophages via the SPP1-ITGB1 axis, leading to CD8^+^ T-cell exhaustion (Tex).

Under the selective pressure of chemotherapy or immunotherapy, the tumor stroma is not a passive bystander; instead, it forms a protective barrier through co-evolution with tumor cells (Figure 4).

1. iCAFs and Paracrine Feed-Forward Loops: In studies of HGSOC, residual lesions following chemotherapy exhibit distinct features of stromal remodeling. Tumor cells under transcriptional stress highly express inflammatory factors *TNF* and *IL-6.* These factors act as paracrine signals, inducing surrounding Cancer-Associated Fibroblasts (CAFs) to transform into inflammatory subtypes (iCAFs). Conversely, iCAFs secrete *IL6*, *CXCL12*, and *LIF*, further activating *JAK/STAT3* and *NF-κB* signaling pathways in tumor cells and enhancing their anti-apoptotic capabilities (e.g., upregulation of *BCL6*), thereby establishing a self-reinforcing “stress-inflammation” feed-forward loop (Zhang K. et al., 2022).
2. Physical and Biochemical Barriers of Activated PSCs: Following neoadjuvant therapy in PDAC, stromal components in residual lesions undergo a significant phenotypic shift. Compared to untreated primary tumors, residual lesions are enriched with activated Pancreatic Stellate Cells (Activated PSCs). These cells specifically express high levels of *RGS5*, *ACTA2* (α-SMA), and the angiogenic factor *VEGFA*, while downregulating typical myofibroblast markers *LUM* and *DCN.* Multiplex immunohistochemistry (mIHC) confirmed that these activated PSCs tightly encase residual malignant ductal cells, not only constituting a physical barrier to drug penetration but also supporting microvascular angiogenesis via the VEGFA-VEGFR2 signaling pathway, thereby providing nutrients for tumor survival (Chen K. et al., 2023). Furthermore, this stromal barrier is accompanied by an enrichment of *SPP1+* macrophages, which interact with *ITGB1* receptors on tumor cells by secreting SPP1, thereby further promoting tumor invasion and drug resistance (Chen K. et al., 2023).
3. Universality of Stromal Heterogeneity: This heterogeneous remodeling of stromal cells is not unique to PDAC. In the integrated analysis of single-cell and Bulk data in bladder cancer, researchers similarly identified multiple CAF subpopulations (such as inflammatory iCAFs and myofibroblastic myCAFs). They found that these subpopulations shape the immunosuppressive microenvironment by secreting factors such as *CXCL12* and *TGFB1* (Shi et al., 2024). Similarly, in the synovial fluid of Rheumatoid Arthritis (RA), fibroblasts exhibit distinct alterations in their differentiation trajectories and form pathogenic interactions with immune cells, suggesting that targeting stromal subpopulations may be a universal therapeutic strategy across diseases (He C. et al., 2025).

### 4.2 Developmental atlases reveal novel myeloid subpopulations: from angiogenesis to neural regulation

The construction of the human developmental immune atlas provides a novel reference frame for analyzing myeloid cell function in disease states. Research has found that many pathological cell states appearing in adult diseases are actually a “recapitulation” or “ectopic activation” of embryonic developmental programs.

1. Proangiogenic Macrophages: Through spatiotemporal single-cell analysis of human embryonic development, Wang et al. identified a specific macrophage subpopulation localized around blood vessels, termed “ PraM”. Originating from the Yolk Sac, this population is characterized transcriptomically by high expression of *VEGFA*, *CXCL8* (IL-8), *IL1B*, and *CD83*, and is localized in perivascular niches across multiple organs (e.g., kidney, skin, heart). Functional experiments demonstrated that PraM supernatant significantly promotes tube formation in vascular endothelial cells, with proangiogenic activity far exceeding that of microglia or other macrophage subpopulations (Wang et al., 2023).
2. Peripheral Microglia-like Cells: Traditional views hold that microglia exist solely within the Central Nervous System (CNS). However, developmental atlas studies unexpectedly identified “microglia-like cells” in fetal skin, testis, and heart that share high transcriptomic similarity (expressing *P2RY12*, *TMEM119*, *SALL1*) and morphological resemblance to CNS microglia. Particularly in the skin, these cells show polar distribution along the dorsal-lateral-ventral axis and make direct physical contact with Neural Crest Cells (NCCs). Experiments proved that depleting these microglia-like cells significantly affects the differentiation of NCCs into the melanocyte lineage, revealing a novel function of myeloid cells in regulating nerve-related precursor cell fates in non-neural tissues (Wang et al., 2023).
3. Subpopulation Shifts in Pathological Environments: This heterogeneity of myeloid subpopulations is particularly critical in disease states. In the subacute phase of Spinal Cord Injury (SCI), the immune microenvironment is dominated by pro-inflammatory M1 macrophages/microglia, thereby impeding neural regeneration (Zhang Q. et al., 2023). However, in Myelodysplastic Syndromes (MDS) and Acute Myeloid Leukemia (AML), single-cell analysis revealed significant transcriptomic heterogeneity in MDSCs, specifically the abnormal activation of inflammatory pathways (such as *CXCL8* signaling), which is closely related to the efficacy of BTK inhibitors (Savardekar et al., 2024).

The discovery of development-associated myeloid subsets—such as PraM and peripheral microglia-like cells—opens novel avenues for precision pharmacology. When these embryonic programs are ectopically reactivated in adult pathological microenvironments, they frequently drive disease progression (e.g., pathological angiogenesis or neuroinflammation). Current single-cell transfer learning frameworks can be powerfully repurposed to address this challenge. By utilizing high-resolution developmental atlases as reference domains, algorithms can accurately map the specific transcriptomic signatures of these reactivated rare subpopulations within complex clinical disease datasets. Consequently, this enables the virtual screening and identification of small-molecule drugs specifically designed to target and reverse these ectopic developmental states without disrupting the surrounding mature, homeostatic immune landscape.

### 4.3 Computational dissection of cell communication networks

To understand how the aforementioned cell subpopulations maintain their niches through molecular signals, computational biology has developed various tools to infer intercellular ligand-receptor interactions.

1. Precise Identification of Receptor-Ligand Pairs: Using computational tools like *sc2MeNetDrug*, researchers can construct intercellular communication networks based on single-cell gene expression data and identify key senders and receivers (Feng et al., 2024). For example, in Multiple Myeloma, tumor cells construct an immune escape microenvironment by upregulating *TGFB1* and interacting with *TGFBR2/3* on stromal cells, or by inhibiting macrophage phagocytosis via the *CD47-SIRPA* axis (Chen M. et al., 2023). In osteoporosis research, network analysis of single-cell data also revealed the core roles of genes such as *CCR4* and *F9* in the interaction between immune cells and Bone Marrow Mesenchymal Stem Cells (BM-MSCs) (Zhang Z. et al., 2022).
2. Coordination of Immune Networks: In studies of leukemia patients receiving Donor Lymphocyte Infusion (DLI), analysis using the DIISCO (Dynamic Integration of Immune Single-Cell Omics) framework discovered that effective anti-tumor responses depend on the synergistic action of immune cell populations. Relapsed patients exhibited decoupling of communication networks between CD4^+^ T cells and myeloid cells, particularly the dysregulation of Interferon (IFN) signaling pathways, which led to leukemia cells evading recognition by downregulating MHC Class II molecules (Maurer et al., 2025).
3. Molecular Anchoring of Spatial Microenvironments: Combining spatial information with single-cell transcriptomic data allows for more precise localization of the physical sites of cell interaction. For instance, in PDAC residual lesions, cell communication analysis found a lack of immune checkpoint interactions such as *PD1-PDL1* between tumor cells and T cells, explaining the mechanism behind “cold tumor” formation. In contrast, the interaction of *SPP1-ITGB1* was locked as a key node maintaining tumor-macrophage crosstalk (Chen M. et al., 2023).

## 5 Pharmacological reprogramming and intervention strategies

Based on pathological subpopulations and microenvironmental interaction networks revealed by scRNA-seq, the paradigm of therapeutic strategy is shifting from simple “cell clearance” to “precise reprogramming” of cell fates. This strategy aims to reverse disease-associated transcriptional states and restore tissue homeostasis and regenerative capacity through specific pharmacological means.

### 5.1 Epigenetic and metabolic reprogramming: reversing cell fates in non-neoplastic diseases

**FIGURE 5:**
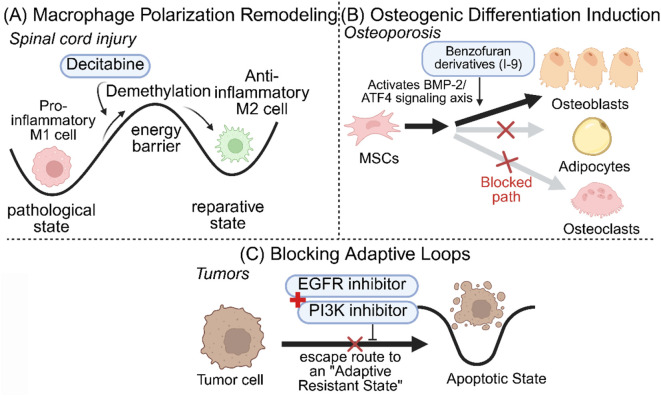
Pharmacological Reprogramming: Epigenetic and Signaling Interventions to Reverse Pathological States. This figure is based on the Waddington Epigenetic Landscape model. **(A)** Macrophage Polarization Remodeling: In spinal cord injury, Decitabine reduces the “energy barrier” via demethylation, facilitating the transition of macrophages from the pro-inflammatory M1 (pathological state) to the anti-inflammatory M2 (reparative state). **(B)** Osteogenic Differentiation Induction: In osteoporosis, Benzofuran derivatives (I-9) activate the BMP-2/ATF4 signaling axis, driving Mesenchymal Stem Cells (MSCs) along the differentiation trajectory toward Osteoblasts rather than Adipocytes or Osteoclasts. **(C)** Blocking Adaptive Loops: In tumors, the combination of PI3K and EGFR inhibitors severs the escape route to an “Adaptive Resistant State”, forcing cells into an “Apoptotic State”.

In the field of regenerative medicine, pharmacological intervention in specific signaling pathways or epigenetic modifications can effectively correct abnormal cell differentiation trajectories (Figure 5).

1. Remodeling Macrophage Polarization in Spinal Cord Injury: Regeneration failure following SCI is primarily attributed to the persistent infiltration of pro-inflammatory M1 macrophages. Zhang et al., using scRNA-seq in combination with network pharmacology, discovered that the DNA methyltransferase inhibitor “Decitabine” exhibits significant immunomodulatory functions. Decitabine does not directly kill immune cells but, through demethylation, downregulates pro-inflammatory genes like *TNF* and *IL1B* while upregulating anti-inflammatory genes like *IL10* and *ARG1*, inducing a transition of macrophages/microglia from the M1 phenotype to the reparative M2 phenotype, thereby improving the microenvironment and promoting functional neural recovery (Zhang Q. et al., 2023). A critical consideration for pharmacological reprogramming is the stability of the induced phenotype following drug withdrawal. Because agents like Decitabine act through epigenetic remodeling (e.g., DNA demethylation), they establish an epigenetic memory that renders the M2 phenotypic transition relatively stable even after the drug is cleared. In acute settings such as SCI, this transient reprogramming intervention is often sufficient to trigger long-lasting tissue repair without the need for continuous dosing (Zhang Q. et al., 2023). It is worth noting, however, that in chronic disease microenvironments characterized by persistent pathological stress, optimized intermittent dosing strategies may be necessary to prevent target cells from reverting to their original pathological states.
2. Inducing Osteogenic Differentiation in Osteoporosis: Addressing the challenge of insufficient bone formation in Senile Osteoporosis (SOP), Zhou et al. utilized scRNA-seq to elucidate the mechanism of action of a novel 6-methoxybenzofuran derivative (Compound 125/I-9). The study showed that this compound activates the BMP-2/Smad signaling pathway in Bone Marrow Mesenchymal Stem Cells (BMSCs) explicitly and induces the expression of the endoplasmic reticulum stress-related factor *ATF4.* This signal reprogramming effectively promoted BMSC differentiation into osteoblasts while inhibiting osteoclast activity, thereby restoring a balance in bone remodeling (Zhou et al., 2023). Additionally, targeting the osteoporosis diagnostic marker *F9*, virtual screening identified the small molecule *DB03742* as a potential targeted intervention drug, further enriching non-hormonal therapeutic options (Zhang Z. et al., 2022).

### 5.2 Blocking adaptive feedback loops: overcoming tumor resistance

In tumor therapy, single-cell pharmacotranscriptomics has revealed compensatory signaling activation in cancer cells under drug pressure, providing a theoretical basis for combination therapy.

1. Feedback Activation Following PI3K/AKT Pathway Inhibition: Targeting HGSOC, Dini et al. developed a multiplexed single-cell screening workflow and found that while PI3K/AKT/mTOR inhibitors (e.g., Dactolisib) suppress the primary pathway, they induce tumor cells to upregulate *CAV1* (Caveolin-1), which subsequently activates RTK, particularly *EGFR* signaling. This “drug-induced feedback loop” is key to resistance. Based on this finding, the combined use of PI3K inhibitors and EGFR inhibitors (e.g., Gefitinib) can synergistically kill resistant subpopulations, improving efficacy by blocking adaptive evolutionary paths (Dini et al., 2025).
2. Heterogeneous Intervention in Myeloid-Derived Suppressor Cells (MDSC): BTK inhibitors (e.g., Ibrutinib) are used to modulate the TME. Single-cell analysis showed that BTK inhibitors can downregulate pro-inflammatory and pro-angiogenic factors (e.g., *CXCL8*, *VEGFA*) in MDSCs. However, MDSCs from different patients exhibit significant heterogeneity in transcriptional response to the drug; in some patients, MDSCs retain strong immunosuppressive features post-treatment, suggesting limitations of single-target interventions and the need for stratified therapy based on patient-specific single-cell characteristics (Savardekar et al., 2024).
3. Chemotherapy-Induced Transcriptional Program Switching: In Multiple Myeloma (MM), VCD chemotherapy regimens cause tumor cells to switch from a high-metabolism/Unfolded Protein Response (UPR) state to a “quiescent/stress” state. Residual clones post-treatment exhibit downregulation of MHC Class I molecules and upregulation of *CD47*, suggesting that drugs, while clearing sensitive cells, select for and induce phenotypes that can escape immune recognition. Targeting key transcription factors such as *YBX1* to address this state transition may represent a new strategy to reverse resistance (Chen M. et al., 2023).

### 5.3 Precision optimization of immune and cell therapies

Single-cell atlases are not only used for screening small-molecule drugs but also directly guide the design and evaluation of immune cell therapies. 1) Atlas-Guided Discovery of CAR-T Targets: In AML, Gottschlich et al. analyzed single-cell transcriptomic atlases of healthy brain and AML samples to find safe targets that avoid neurotoxicity. The study identified *CSF1R* and *CD33* as primary targets and found that although microglia in the brain express these antigens, constructing dual-target Logic-gated CAR-T cells could minimize damage to normal cells in the central nervous system, achieving rational design based on atlases (Gottschlich et al., 2023). 2) Mechanisms of Response to Donor Lymphocyte Infusion (DLI): Addressing DLI therapy for relapsed leukemia patients, Maurer et al., through the DIISCO algorithm integrating longitudinal single-cell data, found that the durability of treatment response depends on synergistic communication between CD4^+^ T cells and myeloid cells in the bone marrow microenvironment, particularly the integrity of the IFN-γ pathway. Relapsed patients exhibited immune network decoupling and downregulation of MHC Class II molecules on leukemia cells, suggesting that re-inducing MHC expression with drugs (e.g., demethylating agents) could be a strategy to potentiate DLI (Maurer et al., 2025). 3) Synovial Microenvironment Remodeling by Anti-TNF/JAK Inhibitors: In Rheumatoid Arthritis (RA), He et al. compared single-cell atlases of synovial fluid before and after treatment with Adalimumab and Tofacitinib. The study found that while both drugs alleviated symptoms, they remodeled monocyte/macrophage subpopulations differently, and some patients showed persistent transformation of fibroblasts towards a pro-inflammatory phenotype post-treatment, providing a molecular explanation for clinical drug switching (He C. et al., 2025).

### 5.4 Computationally driven prediction of drug combinations

Given the cost of experimental screening, the use of computational models to predict drug sensitivity and combination strategies has become an integral part of intervention research.

1. Virtual Drug Screening and Repositioning: Using the SCAD model, researchers can predict cell line responses to drugs at the single-cell level, discovering that certain Head and Neck Cancer cell subpopulations resistant to the Histone Deacetylase (HDAC) inhibitor Vorinostat are sensitive to the EGFR inhibitor Gefitinib, thereby proposing a “synthetic lethality” combination strategy based on subpopulation heterogeneity (Zheng et al., 2023).
2. Gene Targeting to Overcome Cisplatin Resistance: The scDEAL framework predicted through transfer learning that *BCL2A1* and *DKK1* are key resistance driver genes in Cisplatin-resistant oral squamous cell carcinoma cells. Inhibitors or siRNA interference targeting these genes were predicted to effectively reverse the resistant phenotype, demonstrating an efficient path from “algorithm prediction” to “target validation” (Chen et al., 2022).

While small molecules such as Decitabine and Benzofuran derivatives show immense promise in driving macrophage repolarization and osteogenic differentiation, respectively, their clinical translation requires rigorous Pharmacokinetic/Pharmacodynamic (PK/PD) considerations. Inducing pharmacological reprogramming rather than broad cytotoxicity demands precise *in vivo* dosing within a narrow therapeutic concentration window. Furthermore, because agents like Decitabine act via widespread epigenetic modulation (e.g., DNA hypomethylation), evaluating their *in vivo* targeting efficiency and potential off-target epigenetic effects on healthy bystander cell populations is critical. Future frameworks must integrate spatial PK/PD models or leverage targeted delivery systems (e.g., nanocarriers or antibody conjugates) to determine optimal dosing regimens that safely reverse pathological states without disrupting systemic tissue homeostasis.

## 6 Challenges and future perspectives

**FIGURE 6:**
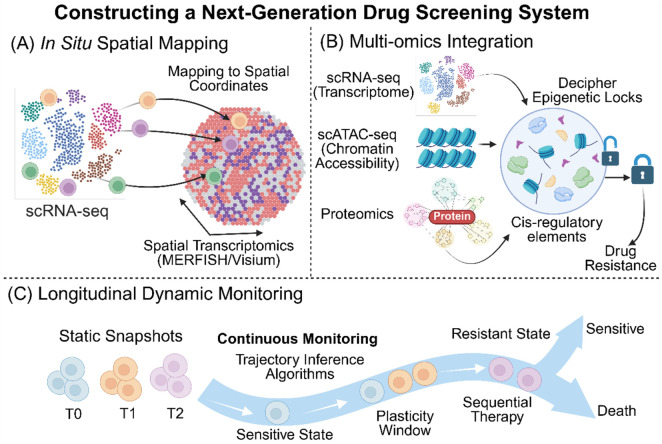
Constructing a Next-Generation Drug Screening System. **(A)** *In Situ* Spatial Mapping: Mapping scRNA-seq data to spatial transcriptomics (MERFISH/Visium) coordinates to identify specific “Resistant Niches”. **(B)** Multi-omics Integration: Integrating scRNA-seq (transcriptome), scATAC-seq (chromatin accessibility), and proteomics data to decipher “Epigenetic Locks” and cis-regulatory elements controlling drug resistance. **(C)** Longitudinal Dynamic Monitoring: Shifting from static snapshots to continuous time-point monitoring, utilizing trajectory inference algorithms to capture the “Plasticity Window” as cells drift from sensitive to resistant states, guiding Sequential Therapy.

Despite the promise of integrating single-cell transcriptomics with DTL, translating these predictions into clinical regimens faces technical and biological hurdles. Future research must address these limitations to develop a robust, spatiotemporally resolved drug-screening system (Figure 6).

### 6.1 From dissociated cells to spatial niches

Current models (e.g., scDEAL) rely on dissociated single-cell suspensions, losing critical spatial information. This limits the assessment of how microenvironmental “neighborhoods”—such as vascular niches or hypoxic cores—influence drug response (Zhang M. et al., 2023). Future frameworks should map drug sensitivity algorithms onto spatial transcriptomic atlases (e.g., MERFISH). Integrating spatial coordinates will enable the identification of specific “Resistant Niches” and differentiate between direct drug targeting and microenvironment-mediated bystander effects (Yao et al., 2023). It is imperative to acknowledge that many spot-based spatial technologies (e.g., Visium) compromise on true single-cell resolution, producing averaged signals that can obscure the detection of extremely rare resistant subpopulations. To overcome this limitation, future frameworks must either rely on imaging-based single-molecule technologies (such as MERFISH, which inherently achieves true single-cell resolution) or employ spatial deconvolution algorithms that use dissociated scRNA-seq data as a reference. These algorithms computationally infer the precise fractional abundance of rare resistant clones within mixed spots, ensuring that the model can identify ‘Resistant Niches' *in situ* without losing the ability to detect rare yet lethal resistant single cells. From a therapeutic decision-making perspective, this lack of spatial context poses a major challenge. If an algorithm fails to account for the physical impedance created by dense stromal barriers, a monotherapy targeting tumor cells may fail clinically due to poor penetrance. Therefore, resolving spatial architecture is crucial for guiding rational combination therapy decisions—such as co-administering stroma-modulating agents (e.g., anti-fibrotics) alongside targeted therapies to ensure adequate drug delivery into ‘Resistant Niches'.

### 6.2 Capturing temporal dynamics and plasticity

Most studies rely on static “snapshots” pre- and post-treatment, making it difficult to distinguish between drug-induced reprogramming and the selection of pre-existing clones (Chen K. et al., 2023). Resistance acquisition is a gradual process with specific “plasticity windows”. Future research requires denser longitudinal sampling combined with trajectory inference algorithms. This dynamic monitoring would enable optimization of sequential treatment strategies, such as intervening with anti-stromal drugs during the early stages of transcriptional stress states to block adaptive resistance loops (Maurer et al., 2025; Zhang K. et al., 2022). At the level of therapeutic decision-making, the reliance on static snapshots hinders the design of effective sequential therapies and adaptive interventions. Without accurately capturing the plasticity window, clinicians cannot determine the exact onset of compensatory resistance feedback loops. Future dynamic monitoring is indispensable for guiding adaptive therapeutic decisions, enabling physicians to pinpoint the optimal temporal window for administering secondary interventions before resistant phenotypes become permanently locked by epigenetic modifications.

### 6.3 Improving biological fidelity via multi-omics

Transfer learning models often depend on cancer cell line databases, which lack the complex TME and cannot simulate stromal-mediated resistance (Chen et al., 2022). To overcome this significant lack of biological context, future computational frameworks must evolve to use patient-derived organoids or microfluidic tumor-on-a-chip models—which preserve stromal and immune cell architectures—as more biologically faithful source domains for algorithm training. Additionally, transcriptomes alone do not reflect the “epigenetic locks” determining future cell potential. To improve biological fidelity, future models must integrate multi-modal data, including scATAC-seq and proteomics. Parsing cis-regulatory elements will help decipher the regulatory logic behind resistance gene expression (Zu et al., 2023). Furthermore, expanding reference datasets beyond oncology to include diseases such as osteoporosis will broaden the applicability of these computational paradigms (He Y. et al., 2025; Savardekar et al., 2024). It is important to note that most current single-cell transfer learning frameworks rely heavily on cancer cell line databases (e.g., GDSC and CCLE), which can inadvertently limit the perceived generalizability of this strategy. Although oncology has spearheaded these efforts due to the urgent clinical challenge of drug resistance, the framework holds immense potential in non-neoplastic diseases, as evidenced by successful applications in rheumatoid arthritis (He C. et al., 2025) and spinal cord injury (Zhang Q. et al., 2023). Future research efforts must prioritize the construction of large-scale, high-throughput pharmacogenomic reference datasets tailored to autoimmune, metabolic, and neurodegenerative diseases. Expanding the source domains beyond oncology will be critical to unleashing the full potential of single-cell pharmacological reprogramming across the broader biomedical spectrum.

### 6.4 Addressing limitations: dataset bias, interpretability, and clinical translation

While the integration of single-cell transcriptomics and deep learning offers a transformative closed-loop strategy for drug discovery, several formidable limitations must be strongly acknowledged. First, dataset bias remains a critical bottleneck. Current Transfer Learning models heavily depend on large-scale 2D cell line databases (e.g., GDSC and CCLE) as their source domains. These *in vitro* models exhibit significant biological biases because they lack the complex immune and stromal architectures of native tissue and often harbor genetic drift from decades of passaging. Furthermore, the clinical scRNA-seq datasets used as target domains frequently exhibit demographic and ancestral biases, potentially limiting the generalizability of predicted pharmacological targets across diverse patient populations.

Second, the interpretability of deep learning algorithms remains a significant hurdle. Neural networks often function as black boxes, extracting high-dimensional mathematical features that are difficult to translate into tangible biological pathways. For clinical pharmacologists to trust AI-driven predictions, these frameworks must incorporate Explainable AI (XAI) modules (e.g., Integrated Gradients) to transparently map “domain-invariant features” to specific, mechanistically sound targets (He Y. et al., 2025). Without clear mechanistic interpretability, predictions risk being driven by statistical artifacts rather than true biological causality.

Finally, bridging the gap toward clinical translation presents immense challenges. *In silico* single-cell transcriptomic predictions do not account for the PK/PD complexities of human physiology. A small molecule that successfully reverses a transcriptional stress state *in vitro* or *in vivo* may fail clinically due to systemic toxicity, rapid metabolic clearance, off-target epigenetic effects, or inability to physically penetrate stromal barriers (e.g., the blood-brain barrier or dense desmoplastic tissue). Therefore, translating these algorithmically defined targets into approved therapies strictly requires rigorous *in vivo* validation and comprehensive toxicological profiling.

### 6.5 Learning from negative outcomes and prediction failures

The current literature on single-cell pharmacology is heavily influenced by publication bias, which favors reporting successful interventions. However, scrutinizing cases where computational predictions fail is equally vital for the field’s advancement. Failures in translating *in silico* predictions to *in vivo* efficacy often stem from the algorithm’s inability to capture the full complexity of the microenvironment. For instance, a transfer learning model may predict high sensitivity of tumor cells to a small molecule, yet the intervention fails *in vivo* because the algorithm does not account for physical stromal barriers—such as those constructed by activated pancreatic stellate cells in PDAC—that prevent drug penetrance (Chen K. et al., 2023). Another major cause of failure is rapid adaptive feedback driven by cellular plasticity; an algorithmically defined single-target therapy may swiftly provoke the activation of compensatory pathways (e.g., EGFR upregulation following PI3K inhibition), thereby turning an initially promising prediction into a negative clinical outcome (Dini et al., 2025). Acknowledging and integrating these failure cases is essential for training next-generation algorithms that can anticipate dynamic feedback loops and physical drug-delivery hurdles.

## 7 Conclusion

The integration of deep transfer learning with single-cell transcriptomics bridges the gap between cell-line pharmacogenomics and clinical data, revealing that therapeutic failure is driven by dynamic transcriptional stress states and stromal barriers rather than genetic mutations alone (Zhang K. et al., 2022; Chen M. et al., 2023). This closed-loop strategy facilitates a paradigm shift from cytotoxicity to “pharmacological reprogramming”, demonstrating that small molecules can reverse pathological cell fates in heterogeneous microenvironments (Dini et al., 2025; Zhang Q. et al., 2023). Ultimately, this AI-driven framework establishes a scalable foundation for discovering non-invasive therapeutic targets and advancing precision medicine.
